# Amplification of photocatalytic degradation of antibiotics (amoxicillin, ciprofloxacin) by sodium doping in nano-crystallite hydroxyapatite

**DOI:** 10.1039/d4ra00126e

**Published:** 2024-04-18

**Authors:** Sakabe Tarannum, Md. Sahadat Hossain, Muhammad Shahriar Bashar, Newaz Mohammed Bahadur, Samina Ahmed

**Affiliations:** a Institute of Glass & Ceramic Research and Testing, Bangladesh Council of Scientific and Industrial Research (BCSIR) Dhaka 1205 Bangladesh shanta_samina@yahoo.com; b Department of Applied Chemistry and Chemical Engineering, Noakhali Science and Technology University Noakhali Bangladesh; c BCSIR Dhaka Laboratories, Bangladesh Council of Scientific and Industrial Research (BCSIR) Dhaka-1205 Bangladesh; d Institute of Energy Research & Development, Bangladesh Council of Scientific and Industrial Research (BCSIR) Dhaka 1205 Bangladesh

## Abstract

In this research, we explain the production of sodium-doped hydroxyapatite (Na_HAp) *via* wet chemical precipitation, followed by crystal modification. To enhance its photocatalytic activity different % of (0.25, 0.5, 1, and 2) sodium doped into HAp crystal. It has been demonstrated that doping is an effective method for modifying the properties of nanomaterials, such as their optical performance and chemical reactivity. Several instrumental approaches were used to characterize this newly synthesized sodium-doped HAp (Na_HAp), *e.g.* scanning electron microscopy (SEM), energy-dispersive X-ray spectroscopy (EDX), X-ray diffraction (XRD), and UV-vis spectrometry were used to analyze the morphology, elemental composition, crystal structure, and optical bandgap, respectively. Under sunlight irradiation, the new Na_HAp photocatalyst was put to use in the process of degrading pharmaceutical pollutants such as antibiotics (amoxicillin and ciprofloxacin). It was found that using a 0.1 g dose of 1% Na_HAp under specified conditions, such as a pH of 7 and 120 minutes of sunlight irradiation, resulted in degradation percentages of 60% and 41.59% for amoxicillin and ciprofloxacin, respectively. Different radical scavengers were utilized to determine the reaction mechanism for the photochemical degradation of antibiotics. Additionally, the ability to be reused and the stability of 1% Na_HAp, a newly developed photocatalyst, were assessed. Therefore, this research adds to our understanding of how to optimize redox capacity for the rapid breakdown of a variety of antibiotics when exposed to sunlight.

## Abstract 2

Sodium-doped hydroxyapatite (Na_HAp) was synthesized for the enhanced photocatalytic degradation of amoxicillin and ciprofloxacin.

## Graphical abstract

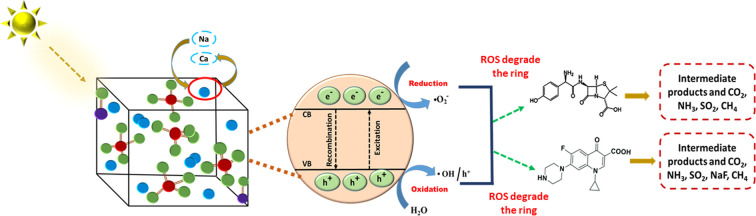

## 1 Introduction

Energy scarcity and pollution are now regarded as crucial environmental issues. Researchers are fascinated by nanomaterials because of their extraordinary characteristics. The research and development of nanomaterials has increased due to their potential applications in electronics, pollution control, healthcare, textiles, agricultural chemicals, and insecticides become more prominent.^1^

Pharmaceutical pollutants are major contaminants in water resources, with their health and environmental effects now being fully acknowledged and evaluated.^2,3^ Toxic levels of antibiotics have been found in natural water sources due to their abuse in animal industries and medicine.^4^ This group includes a wide array of molecules with different physical and chemical characteristics, making their removal from wastewater a complex task.^5,6^ For example, most pharmaceuticals have hydrophobic properties, meaning they don't readily dissolve in water, which makes conventional sorption methods less efficient in removing them.^7,8^

As such, the concentration of residual antibiotics in groundwater and surface water is extremely low (ng L^−1^ to μg L^−1^), and hence doesn't cause an immediate threat to human health or aquatic life. In the meanwhile, many unused antibiotics are being dumped straight into water systems,^9–11^ which poses a risk to aquatic life due to the antibiotics' toxicity and resistance.^12^ Antibiotics, however, can get concentrated and stored in the human body *via* the chain of food intake, which may causes physiological disturbances and even organ illnesses.^13^ Furthermore, bacteria will become resistant to the low concentration of antibiotics due to prolonged exposure, which is a major risk to human and environmental health.^4^ A semi-synthetic β-lactam antibiotic, amoxicillin (AMX) is often used to treat a variety of illnesses in human medicine. One of the fluoroquinolone antibiotics of the second generation is ciprofloxacin (CIP). CIP is one of the most potent antibiotics in the fluoroquinolone category and is used to treat a variety of bacterial illnesses due to its broad-spectrum antibacterial action. Antibiotic overuse, particularly in underdeveloped nations, has led to an increase in problems connected to antibiotic resistance with AMX and CIP-involved therapy.^14,15^ Antibiotics in general, and CIP and AMX in particular, need to be eliminated because antibiotic residues in water can exacerbate the state of antibiotic resistance in some microbes and impair numerous wastewater treatment bioprocesses.

Additionally, because of their inherent biological activity, it is strongly advised to degrade these pollutants either partially or entirely before they are released into the environment. Consequently, a number of methods like adsorption, biofilm, photocatalytic degradation, enhanced oxidation, Fenton, *etc.* have been developed and employed to eliminate antibiotics,^16–18^ with photodegradation appearing as particularly promising.^19^ Among them, photocatalysis involving ultraviolet (UV) or visible light is highly attractive due to its high removal efficiency and environmental friendliness.^20–22^ This approach utilizes a photocatalytic substance, typically a nanoscale semiconductor, capable of producing highly oxidative compounds when exposed to light stimulation.^23^

In recent years, researchers to focus on developing highly effective photocatalysts with properties such as environmental friendliness, inexpensive and significant availability. HAp is an essential mineral found in teeth and bones that has garnered interest from a variety of industries because of its excellent biocompatibility, low cost, minimal environmental effect, and adaptability.^2^ The potential of HAp for application in environmental remediation is demonstrated by the extensive usage of modified HAp to produce HAp-based nano-photocatalysts for the breakdown of organic pollutants in water bodies.^24–27^

Sodium (Na) is widely recognized for its significant impact on biological apatites, possibly due to its involvement in cell adhesion, bone metabolism, and resorption.^28^ Sodium is being found to be a major component of natural skeleton and tooth material, after calcium and phosphorus.^29^

The purpose of this study was to develop a superior photocatalytic material by incorporating sodium into the HAp crystal structure at varying %. This was essential due to the low photocatalytic activity of pure HAp, which limits its utility in industrial settings. The inherent characteristics of both the pure HAp and Na doped HAp were investigated by experimenting with a variety of crystallographic parameters. The assessment of photocatalytic activity involved calculating the rate of antibiotic degradation, including that of amoxicillin and ciprofloxacin, in the presence of sunlight irradiation.

## 2 Experimental

### 2.1 Materials

Purchased from E-Merck Germany, the analytical-grade sodium chloride, calcium hydroxide, and orthophosphoric acid were used without being purified. The laboratory of GRD, IGCRT, BCSIR employed a double distillation process to prepare deionized water (DI).

### 2.2 Preparation of nano-photocatalyst

Hydroxyapatite (HAp) was synthesized by reacting calcium hydroxide and orthophosphoric acid using the wet chemical precipitation method. The appropriate amounts of Ca(OH)_2_ and H_3_PO_4_ were utilized as sources of calcium and phosphorus, respectively, to maintain the molar ratio of calcium and phosphorus at 1.67. A suspension of Ca(OH)_2_ in a water medium was prepared and stirred for 2 hours. After that, a diluted solution of H_3_PO_4_ was gradually introduced drop by drop (at a rate of 3 mL per minute) from a burette into the mixture, maintaining a gentle stirring for uniform mixing of the reactants. The pH level was maintained alkaline (pH = 10–11) by adding ammonia solution. The slurry was allowed to undergo a 24 hour ageing process to complete the reaction, after which the precipitate was separated using a vacuum filter.

The precipitate was dried for two hours at 105 °C in an oven and subsequently introduced into a calcination process for 30 minutes at a temperature of 900 °C (keeping the temperature rising at 3 °C min^−1^). Na doped HAp samples have been produced by adding NaCl to the HAp solution, resulting in the substitution of Ca^2+^ ions with Na^+^ ions within the structure of HAp nanoparticles. The process of doping was carried out using various weight percentages (0.25%, 0.5%, 1%, 2%) of Na^+^ to Ca^2+^. The substance required for the synthesis of nano-Na_HAp was estimated using the charge balancing method outlined in eqn (1).

1 10Ca(OH)_2_ + 6H_3_PO_4_ + *x*NaCl → Ca_(10−*x*)_Na_*x*_(PO_4_)_6_(OH)_2_

### 2.3 Characterization

#### 2.3.1 X-ray diffraction (XRD)

To examine the phases of the powdered samples, a Rigaku SE Smart Lab XRD instrument with a ceramic copper tube (Cu Kα, radiation of *λ* = 1.54060 Å) used as the radiation source. All measurements were done at a constant temperature of 19–20 °C with a diffraction angle range from 5 to 70 of 2*θ* and 0.01 diffraction angle increments. The instrument's operational voltage was kept at 40 kV, while its operating current was kept at 30 mA. Before assessing the samples, the equipment was calibrated to standard silicon. Comparisons were made between the data set and the reference ICDD database (card no. – 01-074-0566).

#### 2.3.2 SEM-EDX

The surface texture and chemical composition of the specimens were evaluated through SEM examination. Specifically, an EVO18, Zeiss machine operating at 25 kV was utilized for this purpose. Elemental composition analysis was conducted using EDX analysis (EDAX, AMETEK, US) with an accelerating voltage of 15 kV.

### 2.4 Measuring the efficiency of photocatalysis

#### 2.4.1 Sunlight-induced degradation of antibiotics

When a photodegradable molecule is exposed to photons, it degrades, particularly at wavelengths often found in light, such as infrared rays, ultraviolet radiation, and daylight. Photocatalytic activity refers to the catalyst's capacity to create pairs of electrons and holes, resulting in the creation of unstable molecules and subsequent additional reactions. Under regulated circumstances, the photocatalytic activity of the produced compounds was evaluated.

Specifically, 0.1 g of each sample was distributed in a 50 mL solution containing amoxicillin and ciprofloxacin at a concentration of 20 ppm. The reaction system was agitated and exposed for a duration of 120 minutes. The antibiotics were subjected to photodegradation studies using sunlight irradiation. Formula (2) was employed to calculate the degradation %, and formula (3) was utilized to calculate the deterioration ability, all based on measurements obtained through an ultraviolet-visible spectrophotometer to evaluate the degradation efficiency.^30^2
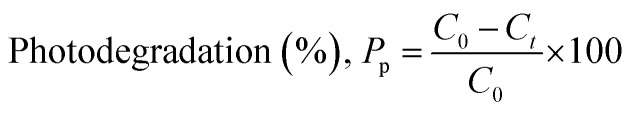
3
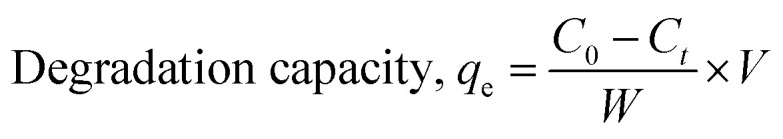


*W* is the weight of the dry sample in grams, *V* is the volume of the experimental solution in liters, and *C*_0_ and *C*_*t*_ stand for the beginning and final concentrations of antibiotics at time *t*, respectively, in both equations. Every piece of data included in this publication has a standard deviation of less than 5%.

#### 2.4.2 Active species capture and study experiments

For the photodegradation of antibiotics, we examined radicals and holes under the same experimental situations in the trapping assays. Before subjecting the solution to sunlight, we mixed in several different scavengers. Three common scavengers were used: ethylenediaminetetraacetic acid (EDTA), sodium oxalate (NaO_2_), and isopropyl alcohol (IPA) to quench h^+^, ˙O_2_^−^, and ˙OH respectively.

## 3 The findings and discussion

### 3.1 Sample characterization

#### 3.1.1 Crystal analysis

**Fig. 1:**
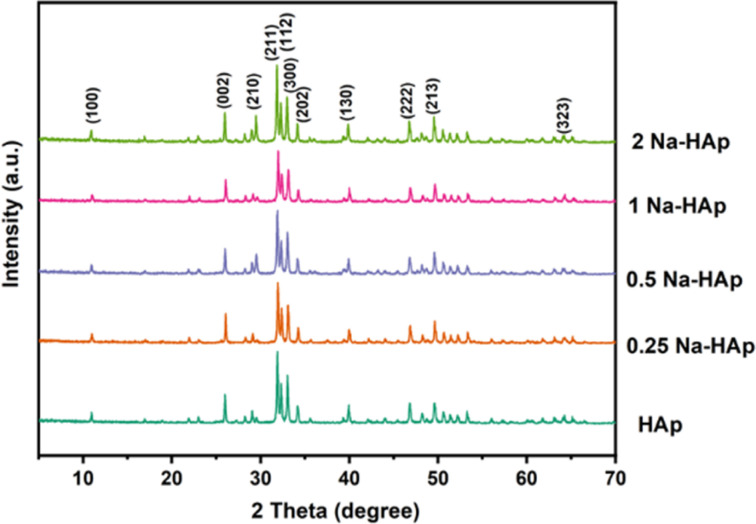
The XRD spectra for pure HAp and Na doped HAp (0.25%, 0.5%, 1%, 2%).

The XRD spectra for undoped HAp and Na doped HAp containing various % of Na (0.25, 0.5, 1, 2) are depicted in Fig. 1. The diffraction angles at 10.94°, 25.98°, 29.07°, 31.92°, 32.31°, 33.04°, 34.18°, 39.95°, 46.8°, 49.6° and 64.25° are indexed to the (100), (002), (210), (211), (112), (300), (202), (130), (222), (213), (323) planes.

Compared to pure HAp, the Intensity of the main peak of HAp (211), (112), (300) decreased slightly for 0.25 and 1 Na-HAp but increased for 0.5% and 2% Na-HAp. A similar trend was also observed for other peaks. Na^+^ is being substituted into HAp, as evidenced by the enhanced and attenuated peaks observed for Na doped HAp compared to pure HAp.

In most cases, no noticeable alterations in the HAp phase are brought about by Na^+^ doping since no secondary phase is identified in the synthesized powder.^29^

The crystallographic analysis was carried out here by computing the lattice parameter equation, crystal size, microstrain, degree of crystallization, crystallinity index, percentage of HAp, cell volume, dislocation density, percentage of β-TCP, and the volume percentage of β-TCP were calculated employing eqn (4)–(12) and shown in Tables 1 and 2.4
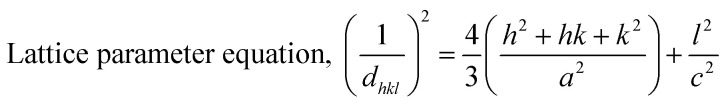
5
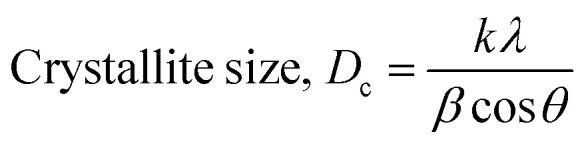
6
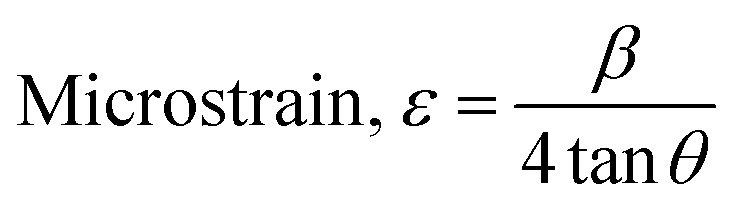
7
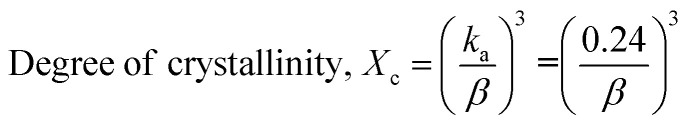
8
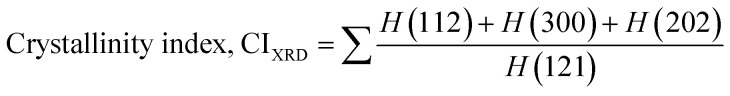

9 Cell volume, *V* = *a*^2^*c* sin 60

10
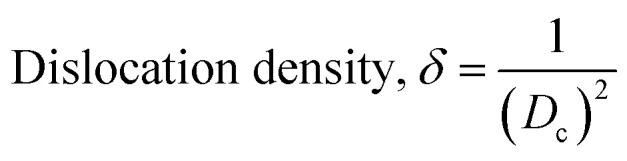
11
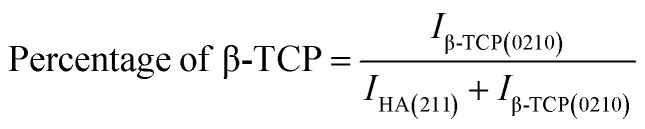
12
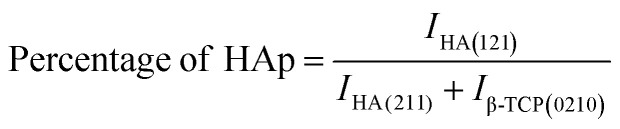


**Table 1:** Shows a comparison between the experimental and reference ICDD databases

Parameter	HAp (ICDD)	Pure HAp	0.25% Na-Hap	0.5% Na-HAp	1% Na-HAp	2% Na-HAp
*h k l*	*d*	*d*	*d*	*d*	*d*	*d*
2 1 1	2.815	2.8043	2.8011	2.8068	2.7969	2.8087
1 1 2	2.778	2.7693	2.7654	2.7715	2.7628	2.7720
3 0 0	2.720	2.7116	2.7064	2.7125	2.7037	2.7155
Lattice parameter	*a* = *b* = 9.42, *c* = 6.88	*a* = *b* = 9.39, *c* = 6.86	*a* = *b* = 9.38, *c* = 6.86	*a* = *b* = 9.40, *c* = 6.86	*a* = *b* = 9.37, *c* = 6.83	*a* = *b* = 9.41, *c* = 6.85
*c*/*a*	0.731	0.728	0.731	0.731	0.729	0.729

**Table 2:** Shows the crystallographic characteristics calculated for the produced samples

Parameters	Pure HAp	0.25% Na-HAp	0.5% Na-HAp	1% Na-HAp	2% Na-HAp
Crystal size (nm)	66.10	70.02	58.59	56.22	64.54
Crystallinity index, CIXRD	2.55	2.52	2.76	2.49	2.65
HAp percentage	100	100	100	100	100
Degree of crystallinity, %	7.08	8.41	4.93	4.35	6.59
β-TCP percentage	0	0	0	0	0
Microstrain, *ε*	0.109	0.103	0.124	0.128	0.112
Dislocation density, (1015 lines per m^2^)	0.23	0.20	0.29	0.32	0.24
Volume of cell	*V* = 522.59	*V* = 522.01	*V* = 524.86	*V* = 518.88	*V* = 525.25

These are the meanings of the symbols used in the equations: *D*_c_ = crystal size; *θ* = degree of diffraction angle; Scherrer's constant *K* = shape factor = 0.94; *a*, *b*, and *c* = lattice parameters; *P* = combined intensity of the HAp (121) reflection and the β-TCP (0210) reflection = 2.275; *h*, *k*, and *l* = planes of the unit cell (in eqn (4)); *H*(*hkl*) = peak height of the (*hkl*) plane; *β* (in radian) = FWHM (full width at half maximum); *K*_a_ = constant = highest value for HAp is 0.24; Iβ-TCP(0210) = the intensity of β-TCP at the (0210) plane and IHA(0210) = HAp; *W*_a_ = the percentage of β-TCP in eqn (11) and volume of cell is denoted as *V*. Incorporating different amounts of Na^+^ into the hexagonal unit cell of HAp replaced different amounts of Ca^2+^ in the structure, which changes its crystallographic properties.

This occurred because Na^+^ ions (1.02 Å) had a larger radius than Ca^2+^ ions (0.99 Å). Table 2 reveals a notable increase in dislocation density with the introduction of sodium into hydroxyapatite. Additionally, the majority of Na-doped nano-materials exhibited a decrease in crystallite size. The observed phenomenon can be related to the decreased crystallinity resulting from the introduction of sodium. The introduction of sodium resulted in an increase in lattice strain. The observed trend in lattice strain may be attributed to the increase in dislocation density resulting from introducing sodium into the hydroxyapatite lattice, as seen in Table 2. Pure HAp has higher crystallinity, larger particulates, and precise stoichiometry, all of which result in low surface reactivity *in vivo*.^31^

NaCl is employed as a doping agent in the crystal structure of HAp, replacing solely Ca^2+^ from the crystal HAp because Na-HAp was synthesized in the basic solution. Sodium has a charge of +1, whereas calcium has a charge of +2, thus when sodium was used to substitute calcium in the hydroxyapatite structure, a hole was formed in the hydroxyapatite structure, resulting in a p-type semiconductor. As a result, a crystal defect forms as a result of the charge difference between Na and Ca, creating a hole. Because of a crystal defect, Na-HAp is more unstable than pure HAp, resulting in increased reactivity.

#### 3.1.2 Rietveld refinement

There was no substantial intense peak combination to help differentiate the phases. To address this issue, Rietveld refinement was used to measure the HAp and β-TCp phases in pure and sodium-doped HAp. This refinement is commonly useful when it is difficult to distinguish the diffraction peaks in a recorded pattern. A Rietveld refinement was used to determine the amount of β-TCp and HAp phases in pure HAp and Na-doped HAp samples. In addition to quantification, lattice parameters *a*, *b*, and *c*, as well as grain size and X-ray density, were revised. The number of points is critical for Rietveld refining; here, 6500 points were evaluated for phase measurement.

The pure HAp was quantified using Rietveld refinement to be 99.80% HAp phase and 0.20% β-TCp phase. In contrast, 0.25_NaHAp had a 99.22% HAp phase and a 0.78% β-TCp phase. The HAp phase % decreased following Na doping in the HAp crystal. As the Na percentage in HAp grows, the HAp phase% decreases compared to pure HAp, confirming Na doping in the HAp crystal. Table 3 contains all of the results of the Rietveld refinement. There was a slight difference in lattice properties between undoped and doped HAp. Rietveld analysis indicated that when the Na concentration increased, the lattice parameters changed and deviated, affecting both the structure and photocatalytic capabilities of the produced samples. This happened because Na^+^ ions (1.02 Å) had a larger radius than Ca^2+^ ions (0.99 Å). The difference in ionic radius of the substituted cations causes deformation of the HAp lattice, resulting in decreased crystallinity and stability while increasing solubility. The grain size of doped HAp crystal fluctuates when compared to pure HAp. The grain size of pure HAp was 95.8, but after 0.25% Na doping, it was increased to 103.3. However, when the doping percentage increased, the grain size decreased, and the lowest for 1% Na_HAp was around 79.6.

**Table 3:** Rietveld refinement data for the synthesized samples

Sample	Hap% or β-TCP%	*a* = *b*	*c*	Grain size	X-ray density
Pure HAP	Hap = 99.80	9.424	6.886	95.8	3.15
	β-TCp = 0.20	10.49	37.75	—	3.003
0.25_NaHAp	Hap = 99.22	9.425	6.884	103.3	3.15
	β-TCp = 0.78	10.51	37.15	—	3.044
0.5_NaHAp	Hap = 99.67	9.426	6.886	90.8	3.15
	β-TCp = 0.33	10.533	37.59	—	2.994
1_NaHAp	Hap = 99.65	9.423	6.887	79.6	3.15
	β-TCp = 0.35	10.54	37.75	361	2.97
2_NaHAp	Hap = 99.75	9.431	6.884	112.4	3.15
	β-TCp = 0.25	10.53	37.68	—	2.988

#### 3.1.3 SEM analysis

**Fig. 2:**
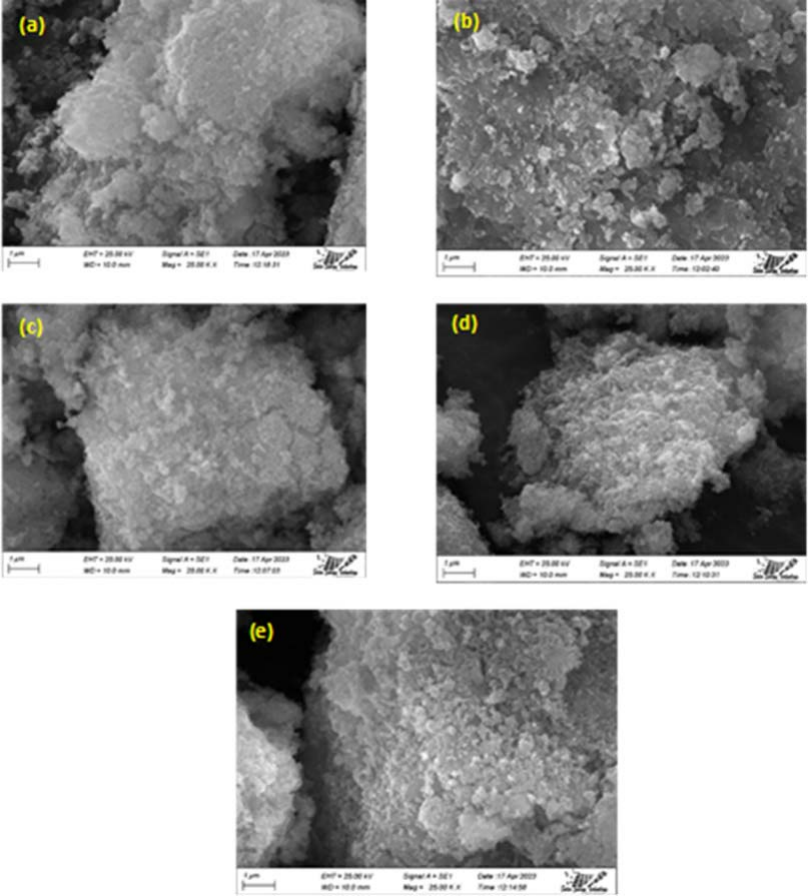
(a–e) Are the scanning electron microscopy images of HAp and 0.25%, 0.5%, 1%, 2% Na-HAp respectively.

Morphological analysis was used to investigate the morphology of the synthesized samples, and the results are shown in Fig. 2. The SEM micrograph of undoped HAp samples revealed more defined hexagonal phases than those of Na-doped HAp samples, but no significant morphological alterations were identified for doping effects. The homogeneity of the materials was apparent in the SEM pictures since no foreign particles were visible. According to SEM images, the integrity of the doped hydroxyapatite particles was less than that of the unmodified HAp. Therefore, doping may result in less crystallinity, which is consistent with XRD.

#### 3.1.4 EDX analysis

**Fig. 3:**
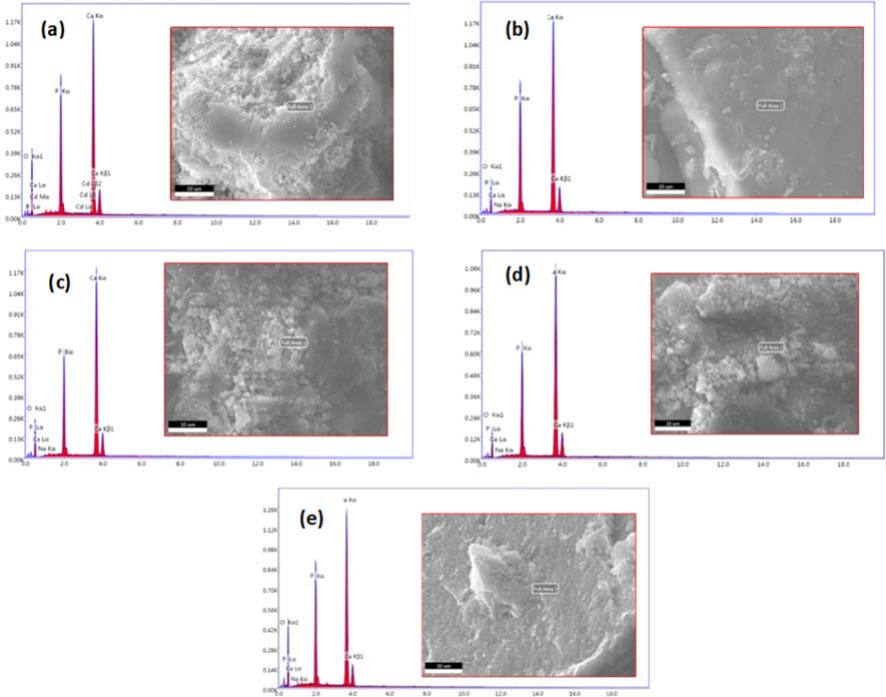
EDX spectra of pure HAp (a) and doped HAp such as 0.25% Na-HAp (b), 0.5% Na-HAp (c), 1% Na-HAp (d), 2% Na-HAp (e).

At the selected spot, the EDX study showed that all of the main elements were present. To ensure that the dopant element Na was distributed consistently among all HAp particles, the analysis steps were repeated. All of the synthesized samples' purity and the existence of Ca, P, O and Na are confirmed by the typical spectra shown in Fig. 3. Moreover, the powder was found to be homogenous in composition.

#### 3.1.5 Band gap

Using an ultraviolet-visible spectrophotometer, the absorption wavelength was used to analyze the optical band gap of both undoped and Na-doped hydroxyapatite (HAp). The optical band gap was calculated using the Tauc technique, which is an equation. In this case, eqn (13) is equivalent to the Tauc method that is applied to get the direct band gap. In this equation, the symbols *α*, *θ*, *E*_g_, *A*, *h* and *n* denote the absorption coefficient, photon frequency, Material's band gap, a constant, Planck's constant, and the power exponent respectively. It is worth noting that the value of *n* is 2 for the direct band gap and 1/2 for the indirect band gap, as indicated by ref. 32 and 33.

13 *αhθ* = *A*(*hθ* −*E*_g_)^*n*^

**Fig. 4:**
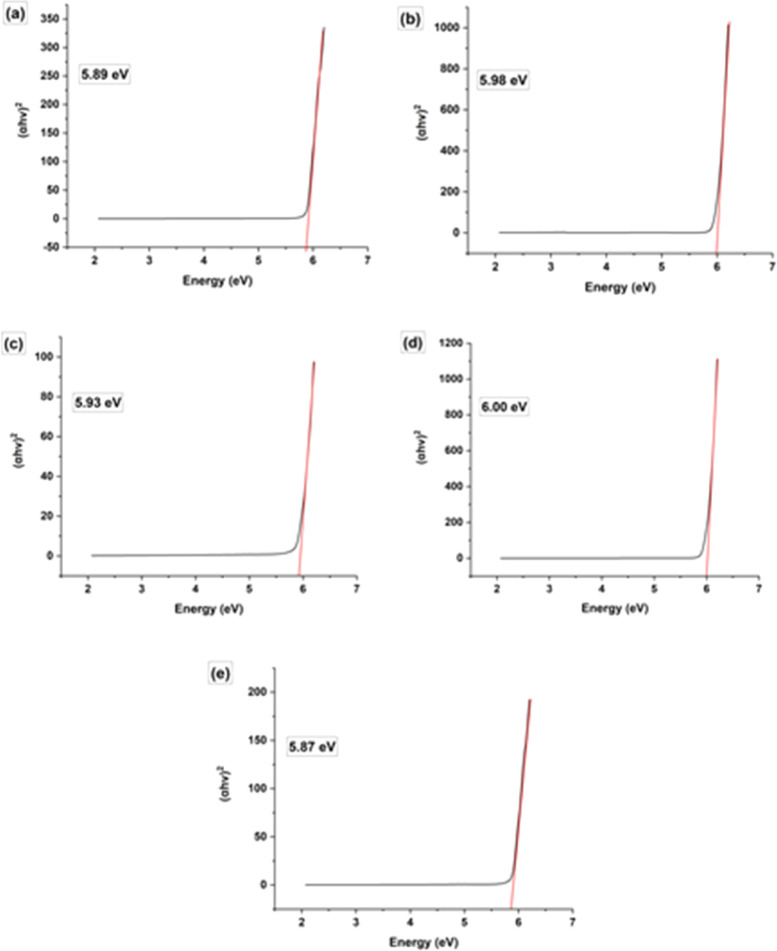
Band gap energy of (a) HAp, (b) 0.25 Na-HAp, (c) 0.5 Na-HAp, (d) 1 Na-HAp, (e) 2 Na-HAp respectively.

The synthesized samples exhibit energy band gap values ranging from 6.00 to 5.87 eV, which exhibit a decreasing order of magnitude: 1 Na-HAp > 0.25 Na-HAp > HAp > 0.5 Na-HAp >2 Na-HAp; Fig. 4 illustrates this. The observed change in *E*_g_ from 5.89 eV in pure HAp to 6.00 eV in the powder resulting from the substitution of Ca^2+^ with Na^+^ (1%) is indicative of an increase in oxygen vacancies originating from –OH and –PO_4_ groups. However, the type of oxygen vacancies generated is distinct when different % (0.25, 0.5, 2) of Na^+^ ions are introduced. Oxygen deficiency in the HAp crystal may have resulted from the introduction of varying % of Na^+^ as a dopant in place of Ca^2+^, according to the experimental data correlating the *E*_g_ shift with the type of vacancies present in the HAp. It may be deduced that the oxygen deficit in the HAp crystal was caused by the introduction of varying percentages of Na^+^ as dopant in the position of Ca^2+^.

### 3.2 Assessment of the undoped HAp and Na doped HAp using photocatalysis

**Fig. 5:**
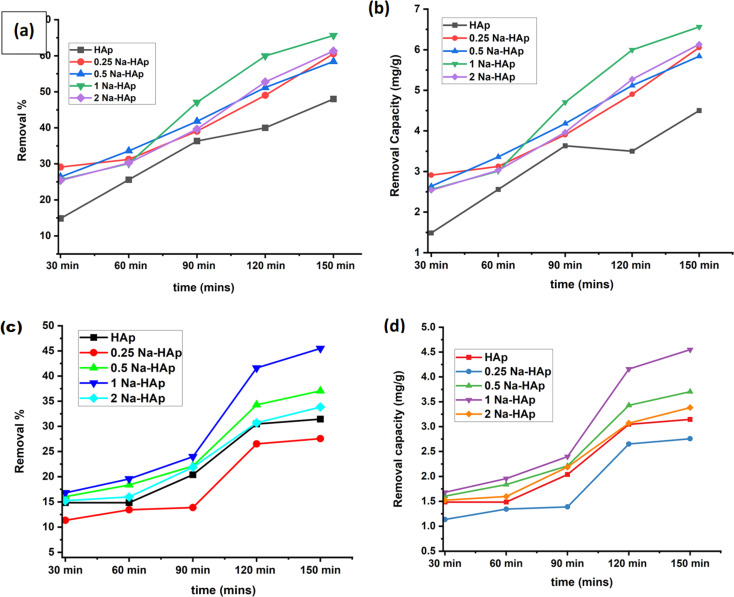
To photo-catalytically break down antibiotics, 1 Na-HAp was optimized in terms of time (a) removal %, (b) removal capacity for amoxicillin, and (c) removal %, (d) removal capacity for ciprofloxacin, respectively.

Amoxicillin and ciprofloxacin degradation were aided using undoped HAp and varying percentages of Na-doped HAp samples. Time is a crucial component of any degradation process since it allows fluctuations in the breakdown % over a predetermined period. 0.1 g of photocatalyst was utilized for up to 150 minutes, and every 30 minutes, the rate of deterioration was measured and noted. The photocatalytic degradation rate and capacity of both antibiotics have been increasing over time, as seen in Fig. 5. Of all the samples, 1% Na_HAp showed the highest rate of amoxicillin degradation, showing 60% after 120 minutes and 65.63% after 150 minutes.

However, after 120 minutes, ciprofloxacin degraded (41.59%) and after 150 minutes, it degraded (45.48%). According to this result, 120 minutes were selected for further experimental study, taking into consideration photocatalytic degradation percentage, capacity, and time. To optimize the efficient sample, a catalyst dose of 0.1 g was administered, sample concentration was 20 ppm and selected time of 120 minutes were used to determine the removal % and capacity for each sample. In comparison to pure Hap, the dopant samples exhibited a higher percentage of removal and capacity.

**Fig. 6:**
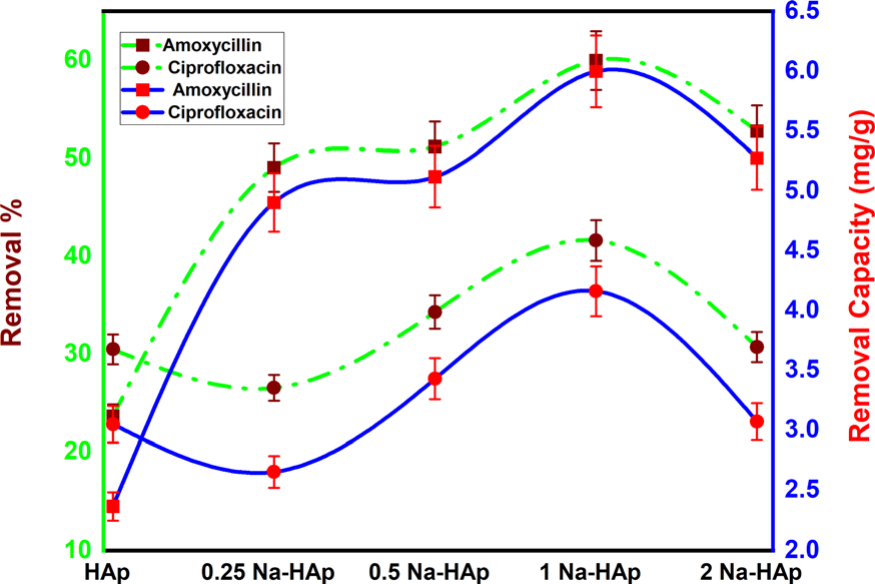
Optimization of efficient sample to degrade amoxicillin and ciprofloxacin.

Out of all the dopant samples examined (Fig. 6), it was observed that the 1% Na_HAp sample exhibited the highest result. The results obtained from the experiment reveal that pure HAp exhibited removal percentages and capacities of 23.6% and 2.4 mg g^−1^ for amoxicillin, and 30.5% and 3 mg g^−1^ for ciprofloxacin, respectively. On the other hand, 1% Na_HAp showed removal percentages and capacities of 59.9% and 6 mg g^−1^ for amoxicillin, and 41.6% and 4.2 mg g^−1^ for ciprofloxacin, respectively.

**Fig. 7:**
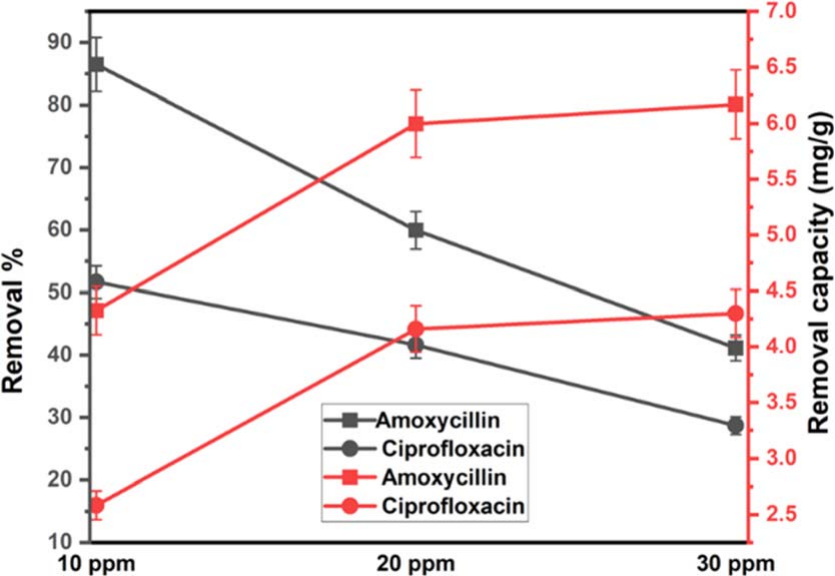
The degradation efficiency of 0.1 g of Na_HAp photocatalyst at various concentrations of amoxicillin and ciprofloxacin.

Less photons reach the catalyst and less reactive species are produced during antibiotic photodegradation when there are more antibiotics present because more antibiotics result in increased activity areas on the catalyst and greater light capture by molecules. Furthermore, additional organic intermediates and end products will be produced, which may compete with the parent antibiotic molecules for the restricted adsorption and photocatalytic areas of the catalyst, thereby inhibiting the breakdown of antibiotics. Here, 0.1 g 1% Na_HAp sample was employed for 120 minutes at both antibiotic's different concentrations, which ranged from 10 to 30 ppm. A decline in the degradation percentage of antibiotic molecules was observed as the initial doses of antibiotics rose. Because of a larger initial dye concentration, the degradation capacity increased even though the degradation percentage decreased shown in Fig. 7.

### 3.3 Reaction mechanism of photocatalysis

In order to help break down antibiotics, holes, free radicals, and electrons can all function as photocatalytic agents. The degradation of antibiotics occurs when they adsorb onto hydroxyapatite (HAp) surfaces. Under sunlight irradiation, HAp generates reactive radicals, such as hydroxyl radicals (˙OH), which initiate antibiotic degradation. Powerful oxidizers can be produced when electrons are stimulated from the valence band to the conduction band, producing vacancies. More reactive species will be produced as long as the photocatalyst is absorbing light since this will prolong the recombination processes of electrons (e) and holes (h^+^). Electron (e) and hole (h^+^) recombination is controlled by the energy levels of the valence band (VB) and conduction band (CB).

According to Mulliken's theory,^34^ this procedure may be mathematically described by eqn (14) and (15).14
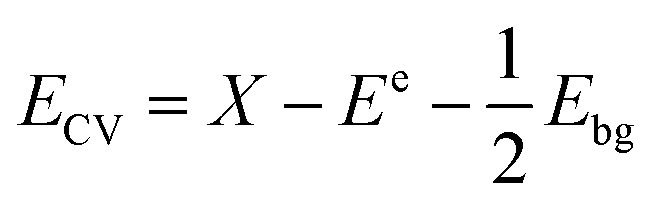

15 *E*_VB_ = *E*_CB_ + *E*_bg_

The symbols *E*_bg_, *E*_VB_, *E*_CB_, *X*, and *E*^e^, denote the band gap energy, valence band energy, conduction band energy, electronegativity of the photocatalyst, and unbound electron energy (value 4.5 eV) respectively. Since only a small quantity of sodium was added to Na-HAp, it was assumed that both undoped HAp and doped HAp had same electronegativity. According to the sources that were reviewed,^34^ the geometric mean of its individual components yielded a value of 5.89 eV. Table 4 displays the calculated valence band and conduction band potentials. The symbols *E*_bg_, *E*_VB_, *E*_CB_, *X*, and *E*^e^, denote the band gap energy, valence band energy, conduction band energy, electronegativity of the photocatalyst, and unbound electron energy (value 4.5 eV) respectively. Since only a small quantity of sodium was added to Na-HAp, it was assumed that both undoped HAp and doped HAp had same electronegativity. According to the sources that were reviewed,^34^ the geometric mean of its individual components yielded a value of 5.89 eV. Table 4 displays the calculated valence band and conduction band potentials.

**Table 4:** Potential of valance band (VB) and conduction band (CB)

Sample ID	VB (eV)	CB (eV)
HAp	4.335	−1.555
0.25% Na_HAp	4.29	−1.6
0.5% Na_HAp	4.315	−1.575
1% Na_HAp	4.28	−1.61
2% Na_HAp	4.345	−1.545

**Fig. 8:**
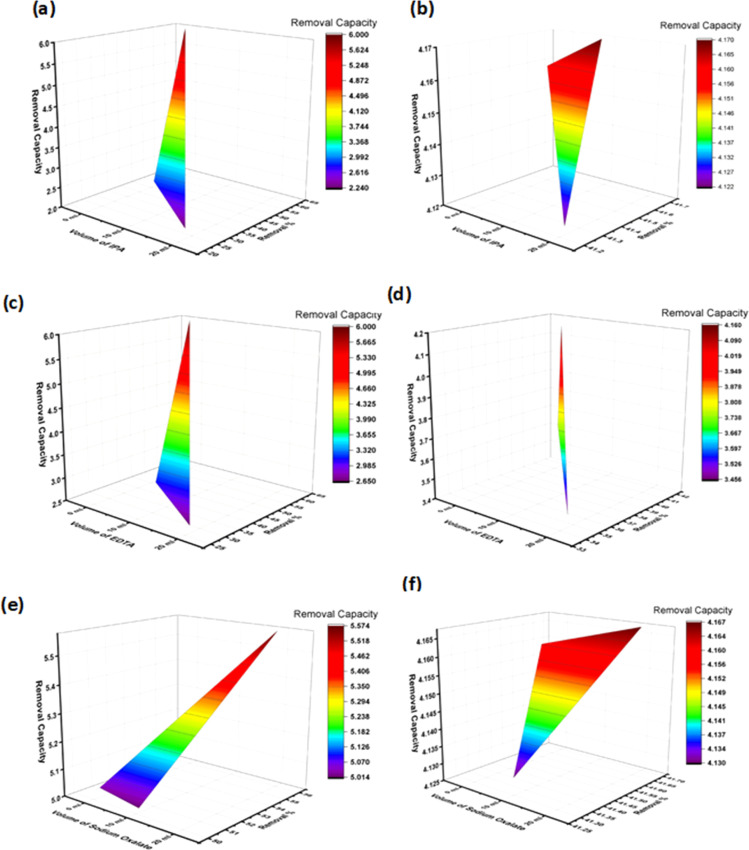
Radical scavenger test on 1% Na-HAp; (a) amoxy-IPA (b) cipro-IPA (c) amoxy-EDTA (d) cipro-EDTA (e) amoxy-sodium oxalate (f) cipro-sodium oxalate.

**Fig. 9:**
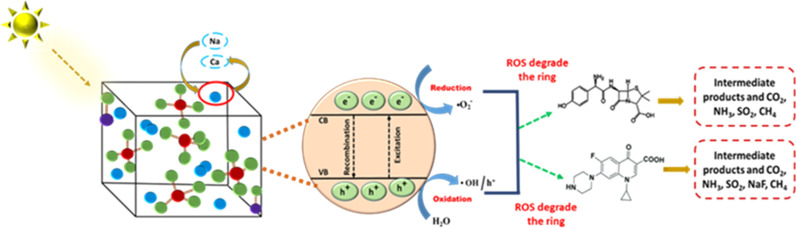
Mechanisms proposed for the photocatalytic degradation of amoxicillin and ciprofloxacin by the sample.

The CB potentials of all the samples were more negative than the redox potential of O_2_/˙O_2_^−^ (−0.33 eV),^35^ while the VB potentials were greater than the OH/˙OH potential (1.99 eV).^35^ This means that both pure and Na_HAp can be used to create radicals for the photocatalysis of antibiotics. Now the radical scavenger lower the photocatalyst's degradation rate and capacity by removing the reactive species (˙OH, h^+^, and ˙O_2_^−^) from the reaction site. As a result, it may explain which reactive species is controlling this particular reaction mechanism. After the absorption of photons by the photocatalyst, three reactive species (˙OH, h^+^, and ˙O_2_^−^) are formed in the photodegradation process. We conducted three sets of radical scavenger experiments for each antibiotic (ciprofloxacin and amoxicillin) for determining the process of degradation. A radical scavenging experiment was conducted using isopropyl alcohol (IPA), EDTA, and sodium oxalate in different volumes (10 mL, 20 mL) individually with 1% Na_HAp photocatalyst. The experiment involved 50 mL of 20 ppm antibiotics, a duration of 120 minutes, and catalyst dose is 0.1 g. The removal % of amoxicillin significantly decreased when IPA and EDTA were added, as compared to sodium oxalate. Moreover, the degradation percentage decreased substantially more with an increase in the amount of IPA and EDTA. Fig. 8a, c and e illustrate the impact of incorporating a radical scavenger on the performance of the sample. Although 59.96% degradation was observed, it dropped to 22.46%, 34.58%, and 44.92%, respectively, when 20 mL of IPA, EDTA, and sodium oxalate were added. Conversely, the degradation % of ciprofloxacin remained unchanged when IPA and sodium oxalate were added. However, the degradation percentage steadily decreased when EDTA was added, as illustrated in Fig. 8b, d and f. A degradation of 41.6% was observed with no scavenger. The elimination percentage reduced to 37.57% when 10 mL EDTA was administered, and 34.58% when 20 mL EDTA was added. Therefore, it may be concluded that ˙OH, h^+^, and ˙O_2_^−^ radicals are accountable for the photodegradation mechanism of amoxicillin, whereas only the h^+^ radical is responsible for the degradation of ciprofloxacin. The following reactions are involved for the degradation of both antibiotics (16)–(24), and Fig. 9 visualizes the reaction mechanism. A comparison is shown in Table 5 showing the degradation of present work and published literature.

16 Ca_10_(PO_4_)_6_(OH)_2_ + *hν* (*λ* > 400 nm) → Ca_10_(PO_4_)_6_(OH)_2_ (e_CV_^−^ + h_VB_^+^)

17 Ca_(10−*x*)_Na_*x*_(PO_4_)_6_(OH)_2_ + *hν* (*λ* > 400 nm) → Ca_(10−*x*)_Na_*x*_(PO_4_)_6_(OH)_2_ (e_CV_^−^ + h_VB_^+^)

18 h_VB_^+^ + amoxicillin (C_16_H_19_N_3_O_5_S) → H_2_O + CO_2_ + degradation products

19 h_VB_^+^ + ciprofloxacin (C_17_H_18_FN_3_O_3_) → H_2_O + CO_2_ + degradation products

20 O_2_ + e_CV_^−^ → ˙O_2_^−^

21 H_2_O/OH^−^ + h_VB_^+^ → ˙OH + H^+^

22 H^+^ + e^−^ → energy

23 Amoxicillin (C_16_H_19_N_3_O_5_S) + ˙O_2_^−^ or ˙OH → H_2_O + CO_2_ + degradation products

24 Ciprofloxacin (C_17_H_18_FN_3_O_3_) + ˙O_2_^−^ or ˙OH → H_2_O + CO_2_ + degradation products

**Table 5:** The photocatalytic efficacy of the 1% Na_HAp materials under sunlight irradiation is compared to that of published studies, and the outcomes are presented

Catalyst	Antibiotics	Reaction condition	Removal (%)	Ref.
1% Na_HAp	Amoxicillin, ciprofloxacin	20 ppm, 0.1 g, 120 min	59.96, 41.59	This work
MoS_2_/ZIF-8	Ciprofloxacin	20 ppm, 20 mg, 180 min	93.2	20
TiO_2_	Ciprofloxacin	160 mg L^−1^, 0.25 g L^−1^, 6 h	96.05	21
ZnO	Ciprofloxacin	80 mg L^−1^, 0.75 g L^−1^, 6 h	100	22
Fe doped ZnO	Ciprofloxacin	10 mg L^−1^, 100 mg L^−1^, 210 min	100	23
TiO_2_	Amoxicillin	17 mg L^−1^, 1.5 g L^−1^, 240 min	84.12	24
WO_3_	Amoxicillin	1 μM, 0.104 g L^−1^, 180 min	99.99	25

### 3.4 Repeatability and stability of the 1% Na_HAp nanoparticle testing

The stability and reusability of the materials are crucial factors in terms of practical use since they directly impact cost reduction in the treatment process. The recycling efficacy of the Na-HAp nanoparticles is evaluated across four successive cycles.

**Fig. 10:**
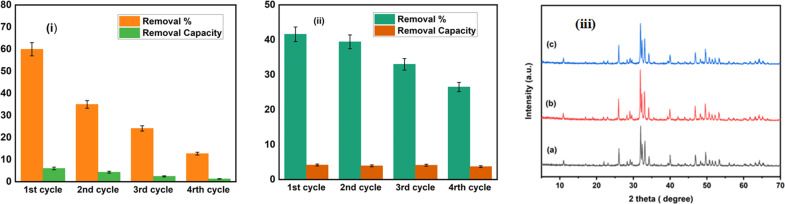
Shows the recycling process used to degrade amoxicillin (i) and ciprofloxacin (ii) using 1% Na_HAp catalyst under sunlight irradiation and (iii) XRD peaks of 1% Na-HAp before (a) and after photocatalysis on amoxicillin (b) and ciprofloxacin (c) respectively.

After 120 minutes of each cycle, the reaction photocatalyst was collected and washed. As a result of photocatalysis, the degradation percentage of amoxicillin dropped from 59.96% to 28.32%, and ciprofloxacin decreased from 41.59% to 26.5% illustrated in Fig. 10. Furthermore, photocatalytic removal capacity exhibited a drop from 5.98 mg g^−1^ to 2.83 mg g^−1^ for amoxicillin and 4.16 mg g^−1^ to 3.7 mg g^−1^ for ciprofloxacin.

This decline in efficiency may have been caused by the loss of photocatalyst during the process of recollecting it as well as the small size of fragmented species that were attached to the photocatalyst materials. Both of these factors contributed to the decrease. Maintaining Na-HAp's original chemical property during photocatalysis is crucial. The XRD patterns of the original 1% Na-HAp and the remaining 1% Na-HAp after four recycling cycles of photodegradation are shown in Fig. 10. There are no unexpected peaks in the XRD data. According to this outcome, it is established that the 1% Na_HAp materials exhibit high stability. On the other hand, the peaks are quite intense after photocatalysis. It is possible that certain antibiotics were absorbed by the Na-HAp nanocrystals during photodegradation, which could be considered for the observed decrease in crystal size (Table 6). Thus, it can be deduced that the stability of the catalyst's structure remained unchanged after its use.

**Table 6:** Presents the results of the crystallographic analysis of 1% Na_HAp performed after the photocatalysis process

Parameter	Before catalysis 1% Na-Hap	After catalysis 1% Na-Hap amoxycillin	After catalysis 1% Na-Hap ciprofloxacin
*h k l*	*d*	*D*	*d*
2 1 1	2.7969	2.8062	2.8011
1 1 2	2.7628	2.7716	2.7663
3 0 0	2.7037	2.7118	2.7059
Lattice parameter	*a* = *b* = 9.37, *c* = 6.83	*a* = *b* = 9.39, *c* = 6.86	*a* = *b* = 9.37, *c* = 6.86
*c*/*a*	0.729	0.731	0.732
Crystallite size, nm	56.21	52.62	48.89
Crystallinity index, CIXRD	2.49	2.63	2.61
HAp percentage	100	100	100
Degree of crystallinity, %	4.35	3.57	2.86
β-TCP percentage	0	0	0
Microstrain, *ε*	0.128	0.137	0.148
Dislocation density, (1015 lines per m^2^)	0.32	0.36	0.42
Volume of cell	V = 518	524	522

## 4 Conclusions

In this study, pure HAp and Na doped HAp samples were effectively synthesized using a simple wet chemical precipitation approach, and the nano-photocatalyst was characterized using SEM-EDX, FTIR, XRD, and UV-vis methods. Under optimal conditions, the catalyst efficiency for the breakdown of amoxicillin and ciprofloxacin antibiotics was evaluated in the presence of sunlight. The results indicated that the 1% Na_HAp catalyst was the most effective when assessed against the other as-prepared photocatalysts. Both amoxicillin and ciprofloxacin respond well to photocatalytic degradation because of the superoxide anions, hydroxyl radicals, and photogenerated holes. Also, after 4th time recycling, it showed good stability and efficiency. The research reported here offers a potential route toward the creation of innovative semiconductor-based photocatalysts with a range of applications.

## Author contributions

Sakabe Tarannum synthesized and characterized the pure and sodium-doped hydroxyapatite, carried out the photocatalytic experiment, and wrote the original manuscript. Md Sahadat Hossain conceived and designed the experiment, analysed the data, and took part in experiment along with characterization with Sakabe Tarannum. Muhammad Shahriar Bashar executed SEM and EDX analysis. Newaz Mohammed Bahadur and Samina Ahmed supervised the findings of this work. Samina Ahmed supervised the overall work and assisted in writing the manuscript.

## Conflicts of interest

There are no conflicts to declare.

## Supplementary Material
